# The Role of Ubiquitination in Osteosarcoma Development and Therapies

**DOI:** 10.3390/biom14070791

**Published:** 2024-07-03

**Authors:** Peng Mao, Zuxi Feng, Yong Liu, Kai Zhang, Guanghai Zhao, Zeyuan Lei, Tianning Di, Haihong Zhang

**Affiliations:** 1 Department of Orthopedics, Lanzhou University Second Hospital, Second Clinical School, Lanzhou University, Lanzhou 730030, China; 2 Key Laboratory of Orthopaedics of Gansu Province, Lanzhou University, Lanzhou 730030, China; 3 Department of Hematology, Lanzhou University Second Hospital, Lanzhou 730030, China

**Keywords:** osteosarcoma, ubiquitination, E3 ligases, E2 ligases, deubiquitinating enzymes, ubiquilins

## Abstract

The ubiquitin–proteasome system (UPS) maintains intracellular protein homeostasis and cellular function by regulating various biological processes. Ubiquitination, a common post-translational modification, plays a crucial role in the regulation of protein degradation, signal transduction, and other physiological and pathological processes, and is involved in the pathogenesis of various cancers, including osteosarcoma. Osteosarcoma, the most common primary malignant bone tumor, is characterized by high metastatic potential and poor prognosis. It is a refractory bone disease, and the main treatment modalities are surgery combined with chemotherapy. Increasing evidence suggests a close association between UPS abnormalities and the progression of osteosarcoma. Due to the complexity and pleiotropy of the ubiquitination system, each step in the ubiquitination process can be targeted by drugs. In recent years, research and development of inhibitors targeting the ubiquitin system have increased gradually, showing great potential for clinical application. This article reviews the role of the ubiquitination system in the development and treatment of osteosarcoma, as well as research progress, with the hope of improving the therapeutic effects and prognosis of osteosarcoma patients by targeting effective molecules in the ubiquitination system.

## 1. Introduction

Osteosarcoma is the most common bone tumor, occurring predominantly in children and adolescents. The primary sites of osteosarcoma are located at the metaphysis of long bones, particularly around the knee joint [1]. The high invasiveness and metastatic rate of osteosarcoma increase the difficulty of treatment and significantly reduce the 5-year survival rate [2]. Prior to 1970, amputation was the main surgical treatment method for malignant bone tumors [3]. However, the functional impairment and low acceptance by patients led limb-salvage surgery to gradually become the preferred treatment for primary bone tumors and bone metastases [4]. Customized prostheses are a feasible option for patients with malignant tumors of the upper limb [5]. Standard treatment for osteosarcoma currently includes wide surgical resection, neoadjuvant chemotherapy, and adjuvant chemotherapy [6]. In recent decades, treatment strategies for osteosarcoma have been continuously optimized, leading to improved survival rates for patients. However, the prognosis for metastatic and recurrent osteosarcoma remains unsatisfactory [7]. Traditional surgical treatments have poor outcomes for advanced osteosarcoma patients, while the combination of surgery and chemotherapy significantly improves patient prognosis [8,9]. Therefore, there is an urgent need to identify molecular mechanisms and biomarkers of osteosarcoma, paving the way for the development of efficient targeted drugs and opening up new treatment strategies for this disease [10].

The UPS is a tightly controlled pathway primarily responsible for clearing short-lived, damaged, and misfolded proteins in the cell nucleus and cytoplasm, as an essential process [11]. The UPS is also a complex biological process, mainly involving the covalent coupling of ubiquitin molecules with substrate proteins, leading to the degradation of substrate proteins through the proteasome pathway [12]. The process of ubiquitin molecules non-covalently binding to substrates is termed ubiquitination, which is catalyzed by a cascade of enzymes consisting of ubiquitin-activating enzyme (E1), ubiquitin-conjugating enzyme (E2), and ubiquitin ligase (E3) [13]. This process of linking substrate proteins with ubiquitin molecules can be reversed by a class of enzymes, removing ubiquitin molecules from substrate proteins, thereby counteracting the effects of substrate protein ubiquitination. These enzymes are known as deubiquitinating enzymes (DUBs) [14]. According to recent research reports, DUBs mainly comprise seven families: the cysteine proteases of the USP (ubiquitin-specific proteases), UCH (ubiquitin C-terminal hydrolases), OTU (ovarian tumor), MJD (Machado–Joseph domain-containing proteases), MINDY (motif interacting with the Ub-containing novel DUB family), and ZUFSP (zinc finger with the UFM1-specific peptidase domain protein) families, and the zinc-dependent metalloproteases of the JAMM (JAB1/MPN/MOV34 domain-associated) family [15]. The USP family has the largest number of members and plays a crucial role in the ubiquitination system. As a type of post-translational modification (PTM), the UPS plays important roles in cellular processes, including apoptosis [16], autophagy [17,18] and the cell cycle [19].

In summary, the UPS regulates intracellular protein homeostasis, and its dysregulation is often closely associated with many human diseases, including various types of cancer [20]. Therefore, a thorough understanding of the UPS mechanism in osteosarcoma cells is crucial for the design and synthesis of small molecule inhibitors or drugs targeting the ubiquitin pathway. Here, we provide a comprehensive review of the current research status and clinical significance of the UPS in osteosarcoma, and discuss the prospects of the UPS in osteosarcoma treatment.

## 2. The UPS

The UPS is one of the crucial pathways for intracellular protein degradation and signal transduction. It mainly consists of three processes: ubiquitination, deubiquitination, and proteasomal degradation. Ubiquitin is a highly conserved protein consisting of 76 amino acids. Ubiquitin molecules can be linked to each other through seven lysine residues (K6, K11, K27, K29, K33, K48, and K63), forming different types of polyubiquitin chains that are attached to substrate proteins, participating in various cellular processes [21]. The ubiquitination process involves the activation of ubiquitin by E1 in an ATP-dependent manner, followed by conjugation of activated ubiquitin to E2, and then E3 mediates the transfer of activated ubiquitin molecules bound to E2 to substrate proteins for tight attachment. Substrate proteins modified by ubiquitination chains of different linkages participate in different cellular activities, such as protein degradation (K48/11/29-linked ubiquitin chains), signal transduction (K63/33-linked ubiquitin chains), DNA damage (K6-linked ubiquitin chains), etc. [22,23]. The proteasomal degradation process involves the recognition and unfolding of polyubiquitinated substrate proteins by the 26S proteasome, leading to their degradation into small peptides [24]. Deubiquitination, on the other hand, is carried out by deubiquitinating enzymes, which remove ubiquitin chains from substrate proteins, thereby stabilizing the substrate proteins and affecting their cellular functions [25].

## 3. E3 Ligases

The ubiquitination process is primarily mediated by three enzymes: ubiquitin-activating enzyme E1, ubiquitin-conjugating enzyme E2, and ubiquitin ligase E3, with the E3 ubiquitin ligase playing a crucial role throughout the process [26]. Interestingly, E3 ubiquitin ligases are involved in early mammalian development and the onset and progression of human diseases. The development of clinical therapies can be propelled by the design and synthesis of specific small molecule inhibitors and drugs targeting E3 ubiquitin ligases [27]. Generally, based on molecular structure and functional mechanisms, E3 ligases can be classified into three main types: homologous to E6-associated protein C-terminus (HECT), really interesting new gene (RING), and RING-in-between-RING (RBR) E3 ligases [28]. Accumulating evidence suggests that E3 ligases play significant roles in the onset and progression of various cancers and have significant implications for enhancing cancer treatment efficacy [28,29,30]. In recent years, E3 ligases have been extensively studied in osteosarcoma, involving numerous signaling pathways and cellular processes. E3 ubiquitin ligases associated with osteosarcoma progression will be discussed in detail below. 

### 3.1. RING E3

#### 3.1.1. The TRIM Protein Family

The tripartite motif (TRIM) protein family is one of the largest subfamilies of E3 ubiquitin ligases, involved in various cellular processes, particularly influencing cancer progression by regulating the cell cycle [31]. Based on the existing research background, it is known that most members of the TRIM family are E3 ubiquitin ligases. TRIM proteins, as RING E3 ligases, covalently modify protein substrates with ubiquitin (Ub), exerting their biological functions [32]. TRIM66 mRNA is abnormally upregulated in osteosarcoma tissues, and its high expression is closely associated with local recurrence, lung metastasis, and low survival rates in osteosarcoma patients. Knockout of the TRIM66 gene inhibits the proliferation, migration, and cell cycle progression of osteosarcoma cells, but induces apoptosis. Additionally, experimental results suggest a positive correlation between TRIM66 and the transforming growth factor-β (TGFR-β) signaling pathway. Moreover, TRIM66 may suppress apoptosis of osteosarcoma cells by downregulating p53 [33]. Research has shown that TRIM7 is highly expressed in osteosarcoma tissues and is an independent risk factor predicting poor prognosis. Research has shown that TRIM7 is highly expressed in osteosarcoma tissues and is an independent risk factor predicting poor prognosis. Mechanistically, TRIM7 directly binds to breast cancer metastasis suppressor 1 (BRMS1) and promotes its ubiquitination and degradation, positively regulating the migration and invasion of osteosarcoma cells. Moreover, elevated levels of TRIM7 in osteosarcoma cells are closely associated with tumor chemoresistance [34]. TRIM11 commonly acts as an oncogene in various cancers, including osteosarcoma. TRIM11 is upregulated in osteosarcoma cells and promotes cell proliferation while inhibiting apoptosis. The oncogenic role of TRIM11 in osteosarcoma cells can be significantly blocked by the extracellular signal-regulated kinase 1/2 (ERK1/2) inhibitor PD98059. At the molecular mechanism level, TRIM11 interacts with dual-specificity phosphatase 6 (DUSP6) and promotes the ubiquitination and degradation of DUSP6, without affecting its transcriptional levels [35]. Jiang et al. found that the high expression level of TRIM46 in OS is closely associated with tumor size, Enneking’s staging, and patient prognosis. At the molecular level, TRIM46, possessing E3 ligase activity, directly binds to peroxisome proliferator-activated receptor alpha (PPARa) and promotes its degradation through the ubiquitin–proteasome pathway. The downregulation of PPARa leads to the activation of the nuclear factor-kB signaling pathway. In terms of cellular function, knockout of the TRIM46 gene significantly inhibits OS cell proliferation and cell cycle progression, inducing apoptosis, whereas overexpression of TRIM46 has the opposite effect [36].

**Figure 1:**
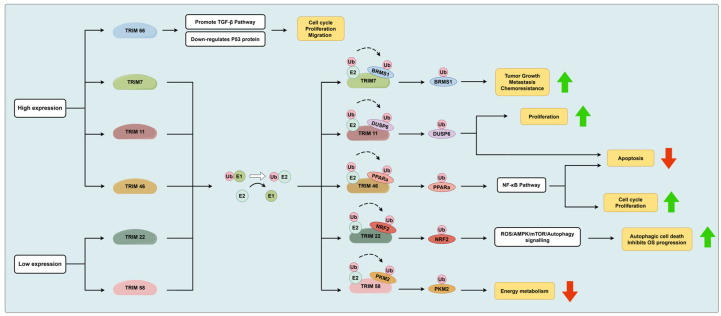
The TRIM family, as E3 ligases, participates in the ubiquitination process of osteosarcoma. In the TRIM family, six molecules including TRIM66, TRIM7, TRIM11, TRIM46, TRIM22, and TRIM58 are involved in the ubiquitination process of osteosarcoma. Among them, TRIM66, TRIM7, TRIM11, and TRIM46 are highly expressed in osteosarcoma cells or tissues, while TRIM22 and TRIM58 are lowly expressed. TRIM66 is involved in the occurrence and development of osteosarcoma by upregulating the TGF-β pathway and downregulating the P53 pathway, with the specific mechanism unclear. TRIM7, TRIM11, TRIM46, TRIM22, and TRIM58 participate in the occurrence and development of osteosarcoma through their role as E3 ligases. In the figure, green upward arrows indicate promotion, and red downward arrows indicate inhibition.

On the contrary, studies have found that some members of the TRIM family are underexpressed in osteosarcoma and act as tumor suppressor genes. TRIM22, not only an ubiquitin E3 ligase but also an interferon-induced protein, has been established to be both nuclear and cytoplasmic in the human osteosarcoma cell line U2OS. Additionally, TRIM22 co-localizes with centrosomes in U2OS cells [37]. Its low expression in osteosarcoma tissues correlates with a favorable prognosis. In vitro and in vivo functional evidence suggests that TRIM22 inhibits osteosarcoma cell proliferation and metastasis. Nuclear factor erythroid 2-related factor 2 (NRF2) is a redox-regulating factor considered as a novel target of TRIM22. TRIM22 interacts with NRF2, inducing its ubiquitination and degradation dependent on TRIM22′s E3 ligase activity. Low levels of NRF2 promote reactive oxygen species (ROS) generation and inhibit mitochondrial membrane potential, thereby activating the AMPK/mTOR signaling pathway. Functional experiments demonstrate that partial knockout of NRF2 reverses the pro-progression effect of TRIM22 knockout in osteosarcoma. Moreover, TRIM22 can inhibit the Warburg effect in osteosarcoma cells. Autophagy activation is observed in osteosarcoma cells overexpressing TRIM22, leading to autophagic cell death. Overall, TRIM22 promotes autophagic osteosarcoma cell death and inhibits osteosarcoma progression by promoting NRF2 proteasomal degradation, activating the ROS/AMPK/mTOR/autophagy signaling [38]. Furthermore, TRIM58 has been found to be underexpressed in human osteosarcoma tissues. Overexpression of TRIM58 significantly inhibits osteosarcoma cell growth and reduces glucose transport and lactate secretion. In osteosarcoma cells, TRIM58 interacts with pyruvate kinase M2 (PKM2) and increases PKM2 polyubiquitination, thereby inhibiting PKM2 expression and activity. Evidence suggests that TRIM58 suppresses osteosarcoma cell progression by negatively regulating energy metabolism in osteosarcoma cells [39]. Figure 1 shows the involvement of TRIM family members in osteosarcoma development with the role of E3 ligases.

#### 3.1.2. MDM2 and CRLs

**Figure 2:**
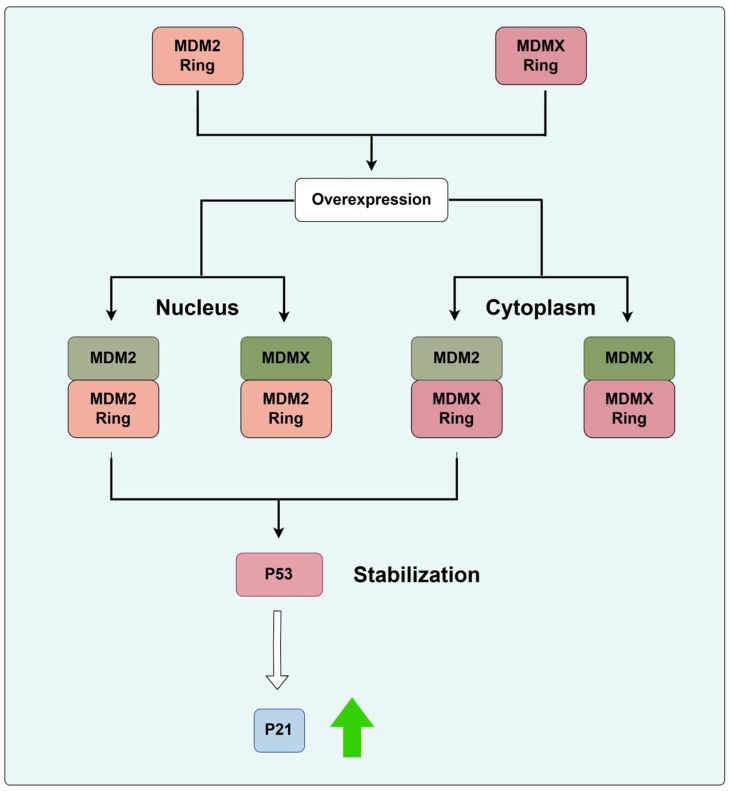
Overexpression of the Mdm2 or MdmX RING domains interfered with the endogenous full-length Mdm2 and MdmX activity and resulted in p53 stabilization and p53 target gene activation. The green arrows in the figure indicate promoting effects.

*Murine double minute 2 (MDM2)* has been extensively studied as an oncogene, with its oncogenic activity primarily associated with the tumor suppressor p53. However, numerous studies have also reported that MDM2 is involved in various non-cancerous human diseases, such as inflammation, neurodegenerative diseases, cardiovascular diseases, kidney diseases, and diabetes [40]. The RING domain at the C-terminus of MDM2 is responsible for its E3 ubiquitin ligase activity, participating in the ubiquitination and degradation process of substrate proteins [41]. It is well known that the E3 ubiquitin ligase MDM2 is one of the antagonists of the tumor suppressor gene p53. The downregulation of p53 is accomplished by Mdm2-mediated p53 ubiquitination and proteasomal degradation through the ubiquitin proteolytic system and by Mdm2 and MdmX-mediated inhibition of p53 transactivation. Recently, Egorova et al. [42] investigated the regulatory role of the RING domain of MDM proteins on P53 in human osteosarcoma (U2OS) cell lines. The study found that the endogenous E3 ligase activity of the MDM2 RING domain is primarily localized in the nucleus, while the MDMX RING domain without endogenous E3 ligase activity is mainly localized in the cytoplasm. Overexpression of the Mdm2 or MdmX RING domains interfered with the endogenous full-length Mdm2 and MdmX activity and resulted in p53 stabilization and p53 target gene activation. Further evidence suggests that the MDM RING domain possesses oncogenic properties independent of p53, emphasizing the distinct structural and functional characteristics of the MDM2 and MDMX RING domains and characterizing their roles in osteosarcoma cell response by disrupting p53-dependent signaling pathways [42] (Figure 2). Overexpression of lncRNA PCAT6 significantly increases the proliferation, migration, and invasion abilities of osteosarcoma cells. Knockdown of the *MDM2* gene inhibits the proliferation, migration, and invasion of osteosarcoma cells. At the molecular level, the overexpression of lncRNA PCAT6 leads to upregulation of MDM2 expression and downregulation of P53 and P21 expression. Additionally, knockout of the *MDM2* gene rescues the downregulation of P53 and P21 caused by the overexpression of lncRNA PCAT6. Therefore, MDM2 mediates the inhibitory effect of lncRNA PCAT6 on the expression of P53 and P21, promoting the proliferation, migration, and invasion of osteosarcoma cells [43]. MDM2 also plays a significant role in cell differentiation. It is known that all-trans retinoic acid (ATRA) induces differentiation of osteosarcoma cells regulated by retinoic acid receptor alpha (RARα), and RARα can be degraded through the ubiquitin–proteasome pathway to inhibit osteosarcoma cell differentiation. Ying et al. discovered that the E3 ubiquitin ligase MDM2 directly binds to RARα and degrades RARα through the ubiquitination–proteasome pathway. Experimental results demonstrate that knockdown of MDM2 upregulates the protein expression levels of RARα, while overexpression of MDM2 promotes the degradation of RARα [44]. Therefore, MDM2 emerges as a potential novel target for differentiation therapy based on all-trans retinoic acid in osteosarcoma. Currently, blocking MDM2 to restore p53 function has become a hot spot in anticancer drug development, with numerous structurally diverse MDM2 inhibitors synthesized and entering relevant clinical trials [45]. Nutlin-3a (nutlin), a small molecule targeting MDM2, interferes with the interaction between MDM2 and P53 in osteosarcoma cells. Removing nutlin from the culture medium in less than 5 min not only triggers ubiquitination of P53 but also dissociates most P53 from its chromatin-binding sites, inhibiting P53 transcription, while P53 has barely begun to degrade at this point. However, when the proteasome inhibitor was applied for several hours, depleting non-conjugated ubiquitin prior to eliminating nutlin, this compromised the removal of DNA-bound p53, as did an E1 ubiquitin ligase inhibitor. This suggests that the ubiquitination of p53 by MDM2 is necessary for its clearance from promoters. Therefore, MDM2 antagonizes p53 not only by covering its transactivation domain and by destabilization, but also by the rapid, ubiquitin-dependent termination of p53–chromatin interactions [46].

**Figure 3:**
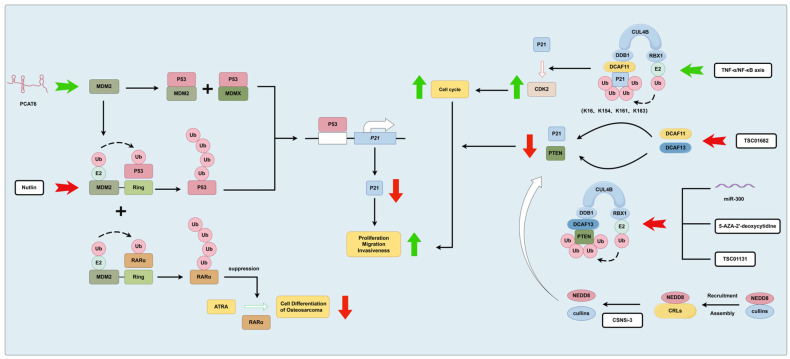
MDM2 and CRLs, as E3 ligases, play a role in the ubiquitination process in osteosarcoma. MDM2 degrades P53 through the ubiquitin–proteasome pathway, and MDM2 and MDMX together mediate the negative regulation of P53 protein expression, thereby inhibiting P21 and promoting the development of osteosarcoma. Moreover, MDM2 can hinder the differentiation of osteosarcoma cells by ubiquitinating and degrading RARα. Additionally, PCAT6 boosts MDM2 expression, while nutlin influences the interaction between the MDM2-RING domain and P53, thereby reducing the ubiquitination and degradation of P53 by MDM2. CRLs degrade P21 and PTEN through the ubiquitination pathway, further fueling the progression of osteosarcoma. Specifically, CRL4B^DCAF11^ targets p21^Cip1^ for ubiquitination at sites K16, K154, K161, and K163, whereas CRL4B^DCAF13^ ubiquitinates PTEN. The TNF-α/NF-κB axis enhances the ubiquitination of p21^Cip1^ by CRL4B^DCAF11^. Conversely, miR-300, 5-AZA-20-deoxycytidine, and TSC01131 inhibit the ubiquitination of PTEN by CRL4B^DCAF13^. TSC01682 impedes the ubiquitination and degradation of P21 and PTEN by downregulating DCAF11 and DCAF13 in CRLs. Additionally, CSN5i-3 disrupts CRLs by cleaving NEDD8 from them, rendering them inactive. Green arrows in the figure signify promotion, while red arrows denote inhibition.

The cullin-RING ubiquitin ligases (CRLs) are a family within the E3 ubiquitin ligase group, which can influence cellular processes related to cancer cell growth and survival pathways by promoting the UPS-mediated degradation of substrate proteins [47]. Cullins are important members of the cullin-RING ubiquitin ligases (CRLs), and cullin 4B (CUL4B) is a key component of the cullin 4B-RING E3 ligase complex (CRL4B). CUL4B, as a member of cullins, facilitates the assembly of E3 ligase complexes and is aberrantly expressed in many cancers, including osteosarcoma. A study found that in human osteosarcoma cells, CUL4B forms an E3 ligase with RBX1 (RING-box 1), DDB1 (DNA damage binding protein 1), and DCAF11 (DDB1 and CUL4 associated factor 11). p21^Cip1^ is a cyclin-dependent kinase (CDK) inhibitor, ubiquitinated specifically by the CRL4B^DCAF11^ E3 ligase at sites such as K16, K154, K161, and K163. Knockdown of any component of the CRL4B^DCAF11^ E3 complex leads to decreased ubiquitination levels of p21^Cip1^, reduced osteosarcoma cell proliferation, and restoration of p21′s inhibitory effect on CDK2, resulting in cell cycle arrest [48]. Additionally, it has been reported that the TNF-α/NF-κB axis negatively regulates the protein levels of p21^Cip1^ by directly targeting CRL4B^DCAF11^, thereby influencing cell cycle progression [49]. Interestingly, the CRL4B^DCAF13^ E3 ligase can also specifically recognize the tumor suppressor factor PTEN (phosphatase and tensin homolog deleted on chromosome 10) and ubiquitinate and degrade PTEN. Aberrant expression of miR-300 or treatment with the DNA methyltransferase inhibitor 5-AZA-20-deoxycytidine significantly reduces the stability of CRL4B^DCAF13^ E3 ligase and the ubiquitination level of PTEN. Functional experiments have shown that a small compound named TSC01131 significantly inhibits the growth of osteosarcoma cells by reducing the stability of CRL4B^DCAF13^ E3 ligase [50]. CRL4B degrades tumor suppressors and cell cycle regulatory factors through the ubiquitin–proteasome pathway, influencing the progression of osteosarcoma. In recent years, extensive research has focused on the assembly process of CRL4B to identify targeted therapies for osteosarcoma treatment. Recently, Chen et al. identified six naturally derived small molecules that specifically disrupt the CUL4B-DDB1 interaction, and found that TSC01682 is the most effective compound among the six small molecules in inhibiting the growth of osteosarcoma cells. Mechanistically, TSC01682 significantly inhibits osteosarcoma cell proliferation and invasion by downregulating DCAF11 and DCAF13 and upregulating CDKN1A (cyclin-dependent kinase inhibitor 1A, also known as p21) and PTEN [51]. Another novel and highly specific JAB1 small molecule inhibitor, CSN5i-3, has been shown to reduce the viability of osteosarcoma cells and has specific effects on the ubiquitin–proteasome system in OS. CSN5i-3 functions by cleaving NEDD8 from cullin-RING ubiquitin ligases (CRLs), rendering them inactive, thus playing a crucial role in protein turnover regulation [52]. Speckle-type POZ protein (SPOP) is an adaptor of the cullin-RING-based E3 ubiquitin ligase complex that is frequently mutated in prostate and endometrial cancers. Research has found that SPOP is underexpressed in osteosarcoma (OS) tissues and cells. Silencing SPOP promotes the migration and invasion abilities of OS cells. At the molecular level, SPOP negatively regulates the PI3K/AKT/NF-κB signaling pathway, making it a potential therapeutic target for OS [53]. The regulation of the P53/P21 pathway by MDM2 and CRLs involved in osteosarcoma development is shown in Figure 3.

#### 3.1.3. RAD18 and RNF8

RAD18 is a RING-type ubiquitin ligase (E3) that plays a crucial role in post-replication repair. It possesses distinct structural domains named RING, UBZ, SAP, and RAD6 binding domain (R6BD) and forms a dimer [54]. Osteosarcoma (OS) is a malignant bone tumor with a high incidence of chemotherapy resistance. Du et al. identified the key gene E3 ubiquitin ligase RAD18 involved in doxorubicin resistance through combined whole-genome CRISPR screening and transcriptome sequencing methods [55]. Previous reports have suggested that the E3 ubiquitin ligase RAD18 participates in cellular DNA damage tolerance mechanisms [56]. RAD18 interacts with meiotic recombination 11 (Mre11), promoting the formation of the Mre11-Rad50-NBS1 (MRN) complex, facilitating activation of the homologous recombination (HR) pathway, ultimately mediating DNA damage tolerance, leading to adverse prognosis and chemotherapy response in OS patients. It is noteworthy that knockout of RAD18 effectively restored in vivo and in vitro chemotherapy responses. Knockdown of RAD18 in combination with doxorubicin significantly increased the anti-tumor activity of doxorubicin. Thus, RAD18 drives doxorubicin resistance in OS by promoting the HR pathway, suggesting that targeting RAD18 is an effective approach to overcoming chemotherapy resistance in OS [55].

**Figure 4:**
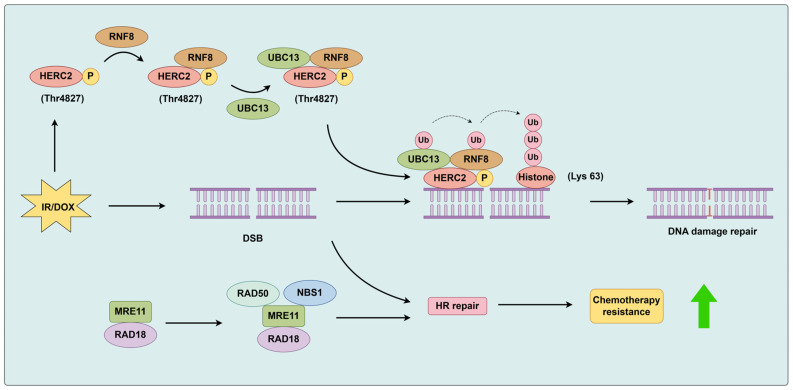
IR induces phosphorylation of HERC2 at Thr4827 and phosphorylated HERC2 forms a complex with RNF8, promoting the interaction between the ubiquitin-conjugating enzyme UBC13 and RNF8, targeting the DSB site, and subsequently facilitating the formation of histone lysine 63-linked ubiquitin chains induced by DNA damage, playing a crucial role in the signaling of damage response. RAD18 interacts with Mre11, promoting the formation of the MRN complex, activating the HR pathway, and ultimately mediating DNA damage tolerance. The green arrows in the figure indicate promoting effects.

The RNF8 (really interesting new gene (RING) finger protein 8) is an E3 ubiquitin ligase associated with DNA damage response, responsible for DNA damage repair and activation of cell cycle checkpoints, thus mainome stability [57]. HERC2 is a factor that regulates ubiquitin-dependent retention of repair proteins on damaged chromosomes. In osteosarcoma U2OS cell lines, ionizing radiation (IR) induces phosphorylation of HERC2 at Thr4827. Phosphorylated HERC2 forms a complex with RNF8, facilitating the recruitment of the ubiquitin-conjugating enzyme UBC13 to RNF8 and targeting the double strand break (DSB) sites. This subsequently promotes the formation of ubiquitin chains linked at lysine 63 of histones induced by DNA damage, playing a crucial role in the signaling of damage response [58,59]. The regulation of DNA damage repair by RAD18 and RNF8 involved in osteosarcoma development is shown in Figure 4.

#### 3.1.4. SIAH1

The E3 ubiquitin ligase SIAH1 contains two domains: an N-terminal RING domain responsible for its enzymatic activity and a C-terminal domain responsible for substrate protein binding [60]. The Wnt/β-catenin signaling pathway plays a crucial role in embryonic development and adult tissue homeostasis [61]. Axin is a concentration-limiting factor responsible for the formation of the β-catenin destruction complex [62]. Ji et al. [63] discovered that in osteosarcoma cells U2OS, the SIAH1 protein interacts with the VxP motif within the GSK3-binding region of Axin, promoting the ubiquitination–proteasomal degradation pathway of Axin and thereby enhancing the stability of WNT-induced β-catenin. However, the effect of SIAH1 on Axin can be counteracted by Axin’s binding to GSK3, and an Axin fragment responsible for binding to SIAH is also involved in binding to GSK3. Dissociation of the Wnt-induced Axin/Gsk3 complex allows SIAH to interact with GSK3-independent Axin and promote its degradation. The SIAH-mediated degradation of Axin is an important feedforward mechanism for sustaining Wnt/β-catenin signaling [63].

#### 3.1.5. Other RING-Type E3 Ligases

CHIP (carboxyl terminus of HSC70-interacting protein) is a cytoplasmic E3 ubiquitin ligase, widely distributed in human tissues and cells, participating in various cellular processes [64]. As is well known, osteosarcoma is a highly invasive malignant tumor with a high rate of lung metastasis, and the efficacy of chemotherapy is not satisfactory. Wang et al. [65] discovered that CHIP mediates the regulatory effect of casein kinase 1α (CK1α) on chromobox homolog 4 (CBX4) expression by promoting the ubiquitination and degradation of CBX4. CK1α phosphorylates CBX4 at threonine 437 to facilitate its turnover through CHIP. Furthermore, evidence suggests that CHIP is highly expressed in osteosarcoma cell lines and tissues, while CK1α expression levels are low in osteosarcoma. At the molecular level, CBX4 recruits GCN5 to the Runx2 promoter, upregulating Runx2 at the transcriptional level, thereby promoting osteosarcoma cell migration and invasion. Interestingly, pyrvinium pamoate (PP), as a selective activator of CK1α, inhibits osteosarcoma metastasis through the CK1α/Cbx4 axis [65].

RLIM (RING finger LIM domain-binding protein) is an ubiquitin protein ligase that targets the CLIM (cofactor of LIM-HD proteins; also known as NLI, Ldb, and Chip) co-factor for degradation via the 26S proteasome pathway [66]. Huang et al. [67] discovered that the E3 ubiquitin ligase RLIM (RING finger LIM domain-binding protein) enhances the response of osteosarcoma U2OS cells to transforming growth factor-β (TGF-β) by directly interacting with the E3 ligase Smad ubiquitination-related factor 1 (Smurf2). Overexpression of RLIM in U2OS cells significantly enhances TGF-β-induced cell migration capacity [67]. Furthermore, Stathmin is an oncoprotein highly expressed in various human malignant tumors, playing a crucial role in maintaining the malignant phenotype. Knockdown of the E3 ubiquitin ligase RLIM upregulates Stathmin protein expression, inhibiting the proliferation and cell cycle progression of osteosarcoma cells. Mechanistically, RLIM interacts with Stathmin and negatively regulates its protein expression levels through the ubiquitin–proteasome pathway [68].

c-CBL (Casitas B-lineage lymphoma) is a RING domain E3 ligase with ubiquitination activity that negatively regulates receptor tyrosine kinase (RTK) signaling by targeting substrate proteins for degradation in lysosomes or by altering the cellular localization of proteins to transmit cellular signals [69]. In recent years, research on the ubiquitin ligase c-Cbl has increasingly expanded in the fields of bone formation and tumorigenesis. c-Cbl can regulate the proliferation, differentiation, and survival of osteoblasts. Moreover, overexpression of c-Cbl suppresses the proliferation of osteosarcoma cells and tumorigenesis [70]. Evidence suggests that low expression of the E3 ubiquitin ligase c-CBL protein is closely associated with high expression levels of epidermal growth factor receptor (EGFR) and platelet-derived growth factor receptor alpha (PDGFRα) in osteosarcoma tissues. Overexpression of c-Cbl significantly reduces the growth, invasion, and migration abilities of osteosarcoma cells. The main mechanism involves the downregulation of receptor tyrosine kinase (RTK), EGFR, and PDGFRα protein expression levels by c-Cbl. In a murine model of bone tumors, the increased expression of c-CBL also decreases RTK expression, leading to reduced proliferation and survival of tumor cells and slowing tumor growth [71].

In 2003, Araki et al. [72] identified ZNRF2 and defined a new family of E3 ubiquitin ligases, known as the ZNRF protein family. ZNRF proteins are uniformly and widely expressed in the brain and peripheral nervous system (PNS), participating in general presynaptic mechanisms shared by both the central nervous system (CNS) and PNS neurons [72]. As is well known, microRNAs (MiRNAs) play crucial roles in processes such as tumor growth, invasion, and migration. Recent studies have found an association between MiRNAs and the UPS. Compared to normal bone tissue, miR-100 is downregulated in osteosarcoma (OS) tissues and is associated with poor prognosis in OS patients. Conversely, a member of the RING superfamily ubiquitin ligase, ZNRF2 shows significantly high expression levels. Overexpression of ZNRF2 or knockdown of miR-100 can promote the growth of OS cells and increase cell survival rates after treatment with doxorubicin. A possible mechanism is that miR-100 binds to the 3′ untranslated region of the ZNRF2 gene, thereby preventing its protein translation [73].

### 3.2. HECT E3

WW domain-containing E3 ubiquitin protein ligase 1 (WWP1) is a HECT-type E3 ubiquitin ligase containing WW domains, participating in various cellular biological processes such as protein degradation and signal transduction [74]. WWP1 is implicated in the pathogenesis of various human diseases, particularly playing a significant role in the onset and progression of tumors. The expression of WWP1 in osteosarcoma tissues is markedly higher than in their matched normal bone tissues. Knockdown of WWP1 significantly inhibits the growth and invasion of osteosarcoma cells, leading to G1 phase arrest and apoptosis. At the molecular level, the protein levels of apoptosis-related proteins (Bcl2, bax) and invasion-related factors (MMP2, MMP9, β-catenin, E-cadherin) also undergo corresponding changes upon WWP1 knockout [75]. Malignant bone tumor osteosarcoma (OS) is closely associated with its stem cell properties, recurrence rate, and drug resistance. Zhang et al. [76] discovered a novel osteosarcoma tumor suppressor gene, *UEV1A*, which negatively regulates the stem cell properties of OS cells and induces terminal differentiation by affecting the BMP signaling pathway. The specific mechanism involves UEV1A interacting with Smad1 and promoting the ubiquitination degradation of Smad1 via UBCH5B-SMurf1. Functionally, the overexpression of UEV1A significantly inhibits the proliferation capacity of OS cells and enhances sensitivity to chemotherapy drugs [76]. Additionally, the direct binding of E3 ligases Smad ubiquitination-related factor 1 (Smurf2) with E3 ubiquitin ligase RLIM (RING finger LIM domain-binding protein) markedly enhances the response of osteosarcoma U2OS cells to TGF-β (transforming growth factor-β) [68].

## 4. E2 Ligases

UBCH10 is a cancer-related E2-ubiquitin-conjugating enzyme. Past studies have shown a significant correlation between highly expressed UBCH10 and tumor grading and progression. One study demonstrated that knocking down UBCH10 in osteosarcoma cell lines resulted in the downregulation of Ki-67, MMP-3 (matrix metalloproteinase-3), and MMP-9 (matrix metalloproteinase-9), leading to decreased proliferation, invasion, and migration capabilities of OS cells [77]. Ubiquitin-conjugating enzyme E2T (UBE2T) is a member of the E2 family and is highly expressed in various cancers. One study found that UBE2T is also aberrantly overexpressed in osteosarcoma tissues and cell lines. UBE2T promotes the proliferation, migration, and invasion of osteosarcoma cells through positive regulation of the PI3K/Akt signaling pathway, suggesting that UBE2T may serve as an important target in osteosarcoma therapy [78]. Research has shown that the expression levels of NEDD8-activating enzyme E1 (NAE1) and ubiquitin-conjugating enzyme E2M (UBE2M) are significantly higher in osteosarcoma tissues and cells compared to normal bone tissues and cells, potentially contributing to the onset and progression of osteosarcoma. Treatment of osteosarcoma cells with the anticancer inhibitor MLN4924 inhibits the degradation of cullins, inducing the accumulation of several tumor-suppressive substrates of cullin-RING E3 ubiquitin ligases (CRLs), including CDT1, Wee1, p21, p27, Noxa, and p16. This leads to DNA damage in osteosarcoma cells, resulting in decreased cell survival, accelerated cellular senescence, and apoptosis [79]. The clock gene Aryl hydrocarbon receptor nuclear translocator-like protein 1 (*Arntl* or *BMAL1*) interacts with an E3-independent E2 ubiquitin-conjugating enzyme, E2 O (UBE2O). UBE2O negatively regulates the protein levels of BMAL1 by promoting its ubiquitination and degradation. Additionally, knockout of UBE2O increases BMAL1-mediated transcriptional activity without altering BMAL1 gene expression. Bioluminescence experiments demonstrate that *UBE2O* gene knockout enhances the circadian rhythm amplitude in human osteosarcoma U2OS cells. This study proves that UBE2O is a critical regulatory factor in the ubiquitin–proteasome system, modulating the transcriptional activity and circadian rhythm function of BMAL1 by promoting its ubiquitination and degradation [80].

## 5. DUBs

Like most cellular processes, ubiquitination is reversible. There exists a class of enzymes that target substrate proteins and catalyze the removal of Ub or polyubiquitin chains from substrate proteins, collectively known as deubiquitinating enzymes (DUBs). Aberrant expression of DUBs is associated with many human diseases, including cancer, and targeting DUBs has provided new insights and directions for anticancer therapy [81]. A list of DUBs involved in osteosarcoma is provided in Table 1.

### 5.1. USP1

Among all human DUBs, ubiquitin-specific protease 1 (USP1) is best characterized. USP1 is involved in crucial physiological and pathological processes such as cellular DNA damage response, autophagy, and cell differentiation. USP1 is aberrantly expressed in various human cancers and participates in tumor initiation and progression, holding significant implications for targeted cancer therapy. USP1 promotes the proliferation of pancreatic ductal adenocarcinoma (PDAC) cells, BRCA1-deficient tumors cells, and glioblastoma cells [106,107,108]. In 2011, Williams et al. [82] first reported the role of USP1 in osteosarcoma progression. The ubiquitin–proteasome pathway degradation of inhibitors of DNA binding (IDs) plays a crucial role in inhibiting differentiation and maintaining stem cell fate in differentiated tissues. However, in tumors, the ubiquitination and proteasome degradation of IDs are largely abolished. USP1 binds to IDs (ID1, ID2, and ID3), leading to their deubiquitination and stabilizing the protein expression of IDs. Knockdown of USP1 in osteosarcoma cells downregulates the expression levels of ID proteins, inhibits the cell cycle, and osteogenic differentiation. Conversely, overexpression of USP1 in mesenchymal stem cells stabilizes ID proteins, inhibits osteoblast differentiation, and promotes proliferation. These studies suggest that USP1 maintains the stem cell state of osteosarcoma and may be an important target for differentiation therapy [82]. A study revealed that USP1 is highly expressed in osteosarcoma tissues, and genetic knockout of USP1 significantly inhibits the growth and invasion capabilities of osteosarcoma cells. Importantly, knockdown of USP1 downregulates the expression of several proteins associated with the onset and progression of tumors, including SIK2, MMP2, GSK-3β, Bcl2, STAT3, Cyclin E1, Notch1, Wnt-1, and Cyclin A1 [83]. Abundant research findings indicate that microRNAs (miRNAs/miRs) are closely associated with the ubiquitin system and play important roles in the progression of various cancers. Studies have shown that miR-192-5p is downregulated in osteosarcoma tissues and cells, while USP1 is aberrantly overexpressed. miR-192-5p inhibits the proliferation, invasion, migration, and apoptosis of osteosarcoma cells by negatively regulating the expression of USP1. Additionally, overexpression of miR-192-5p significantly enhances the sensitivity of osteosarcoma cells to cisplatin. Therefore, miR-192-5p may serve as a valuable biomarker, and the miR-192-5p/USP1 axis could be a new therapeutic target for osteosarcoma treatment [86]. USP1 influences osteosarcoma progression by regulating the Hippo signaling pathway. Elevated levels of USP1 expression in osteosarcoma (OS) are closely associated with maintaining the mesenchymal stem cell state. Yuan et al. discovered that, in OS cell lines, USP1 directly binds to the transcriptional coactivator with PDZ-binding motif (TAZ) and promotes TAZ deubiquitination, thereby enhancing Hippo signaling pathway function. Functionally, TAZ overexpression partially reverses the anti-tumor effects of USP1 knockdown or ML323 (a selective inhibitor of USP1) treatment [84]. ML323 is a highly potent inhibitor of the USP1-UAF1 deubiquitinase complex. In osteosarcoma and non-small cell lung cancer cells, ML323 inhibits the deubiquitination activity of the USP1-UAF1 complex in the DNA translesion synthesis and Fanconi anemia pathways in response to DNA damage. Additionally, ML323 significantly inhibits cell viability and enhances the cytotoxicity of cisplatin-resistant cells to cisplatin [85]. In summary, USP1 plays an oncogenic role in osteosarcoma and represents a potential target molecule for osteosarcoma treatment.

### 5.2. USP4/USP17

USP4 was the first reported nuclear-cytoplasmic shuttling protein, exhibiting differential subcellular localization and significant functional diversity across various cell types [109]. USP4 is widely expressed in most normal tissues and cells, but shows high expression in skeletal muscle, the heart, the pancreas, and the testis [110]. USP17 exhibits inconsistent expression in different tumors and plays crucial roles in cellular processes such as proliferation, apoptosis, and migration [111]. USP17 expression is upregulated in osteosarcoma tissues and cells, and its overexpression promotes the proliferation, migration and invasion of osteosarcoma cells. At the molecular level, USP17 directly interacts with SMAD4 and stabilizes it through its deubiquitinase activity. Consequently, USP17 enhances osteosarcoma progression by stabilizing SMAD4 [88]. USP4 and USP17 synergistically interact in multiple tumor cells [87,112]. Sarri et al. [87] confirmed that in osteosarcoma cells, USP4 and USP17 significantly inhibit the ubiquitination of platelet-derived growth factor receptor β (PDGFRβ) and are able to remove polyubiquitin chains linked to Lys63 and Lys48 on the receptor. Deubiquitination of PDGFRβ by USP17 and USP4 regulates its transport rate but does not affect the stability of PDGFRβ. USP17 prolongs the presence of the receptor on the cell surface, while USP4 affects the rate of transport to early endosomes. Deubiquitination of PDGFRβ mediated by USP17 and USP4 leads to dysregulated downstream signaling of STAT3 transduction and transcription, impacting osteosarcoma cell proliferation [87].

### 5.3. USP6/USP27X/USP41/USP43

Ubiquitin-specific proteases (USPs) play a crucial role in the onset and progression of many cancers, and osteosarcoma (OS) is no exception. RNA sequencing analysis of mesenchymal stem cells differentiated or undifferentiated into osteoblasts, derived from OS cells, revealed increased expression of four USPs: USP6, USP27x, USP41, and USP43. Tissue microarray analysis of patient biopsy specimens showed upregulation of these four USPs at the protein level. Additionally, Kaplan–Meyer analysis indicated that the high expression of USP6 and USP41 correlates with lower patient survival rates. Experimental evidence suggests that the USP inhibitor PR619 enhances protein ubiquitination in OS cells, inhibiting tumor growth and lung metastasis in OS tumors [113].

### 5.4. USP7

Ubiquitin-specific protease 7 (USP7) belongs to the deubiquitinase family and participates in the malignant processes of various cancers by targeting key cancer proteins. Compared to paired adjacent normal tissues, USP7 is significantly upregulated in osteosarcoma (OS) tumor tissues, and high expression levels of USP7 are positively correlated with TNM staging and metastasis in OS patients [91]. USP7 directly binds to β-catenin and activates the Wnt/β-catenin signaling pathway, promoting the migration and invasion of OS cells [91]. In addition, mTOR complex 1 (mTORC1) is involved in the occurrence and development of osteosarcoma by regulating the stability of USP7 mRNA. Specifically, mTORC1 induces m6A modification in the coding sequence (CDS) region of USP7 to stabilize its mRNA. USP7, in turn, enhances the NLRP3 inflammatory signaling pathway promoting proliferation and migration of OS cells by reducing ubiquitination of K48 linked NLRP3 [90]. Micafungin has been found in drug studies to induce apoptosis in osteosarcoma cells and prevent epithelial–mesenchymal transition (EMT) in a USP7/AKT/GSK-3β pathway-dependent manner, exerting anti-tumor effects [89].

### 5.5. USP9X

Increasing evidence suggests that USP9X plays a critical role in human normal development and disease processes, particularly in terms of its impact on cancer [114]. A recent study found that the deubiquitinase USP9X stabilizes the oncogene SOX2 protein expression through its deubiquitinating activity, and SOX2 mediates the promoting effect of USP9X on osteosarcoma cell proliferation [92]. It is noteworthy that naringenin (NGA), an effective component extracted from Ginkgo biloba leaves, significantly inhibits osteosarcoma cell proliferation by directly targeting the USP9X-SOX2 axis [92]. In addition, USP9X is also involved in the proliferation, migration, and invasion of osteosarcoma by regulating the ERK1/2 and PI3K/Akt signaling pathways [93].

### 5.6. USP11

In the human osteosarcoma U2OS cell line, knockdown of the deubiquitinase USP11 activates the DNA damage response (DDR) in undamaged cells and enhances sensitivity to PARP inhibition, ionizing radiation, and other insults [94]. Additionally, evidence suggests that USP11 facilitates homologous recombination (HR) repair [94]. Furthermore, in U2-OS cell lines, USP11 deubiquitinates RAE1, playing a critical role in bipolar spindle formation [95].

### 5.7. USP22

Ubiquitin-specific protease 22 (USP22) is a member of the deubiquitinase family. Zhang et al. reported high expression of USP22 in osteosarcoma tissues and cells. USP22 influences osteosarcoma cell proliferation, invasion, and migration by regulating the PI3K/Akt signaling pathway and the expression of EMT-related markers [97]. Another study demonstrated the low expression of miR-140 and p21, and high expression of USP22 and LSD1 in osteosarcoma cells [96]. Overexpression of miR-140 inhibits osteosarcoma cell proliferation, migration, and invasion and promotes apoptosis by downregulating USP22 expression. USP22 inhibits LSD1 ubiquitination and degradation through deubiquitination, thereby promoting osteosarcoma progression. Therefore, the miR-140/USP22/LSD1 axis regulates osteosarcoma tumor progression [96]. Other research has explored mRNA methylation epigenetic changes beneficial for the development of targeted therapies in osteosarcoma growth. In osteosarcoma patients, RNA demethylase ALKBH5 is overexpressed and promotes osteosarcoma growth and metastasis. ALKBH5 upregulates the expression of USP22 and RNF40 by regulating the m6A levels of histones, leading to inhibition of H2A monoubiquitination and induction of critical oncogenes, ultimately promoting osteosarcoma progression [98]. Long non-coding RNA (lncRNA) has been shown to participate in the development and progression of osteosarcoma. Chen et al. [99] elucidated the important role of linc00265 in osteosarcoma. Overexpression of linc00265 promotes osteosarcoma cell proliferation, migration, and invasion. Importantly, linc00265 exerts its oncogenic function by regulating the expression levels of miR-485-5p and USP22 in osteosarcoma [99].

### 5.8. USP39

USP39 is overexpressed in various cancers, promoting cancer progression. Bioinformatics analysis based on the publicly available Oncomine database revealed higher mRNA expression and DNA copy numbers of ubiquitin-specific peptidase 39 (USP39) in osteosarcoma cancer tissues compared to normal tissues [100]. Protein imprinting analysis also indicated aberrant endogenous expression of USP39 in different osteosarcoma cells. Knockdown of USP39 in osteosarcoma cells resulted in reduced cell proliferation, cell cycle arrest, and induction of apoptosis. Mechanistic studies revealed that USP39 inhibits the osteosarcoma cell cycle through a p21-dependent pathway and promotes osteosarcoma cell apoptosis through PARP cleavage [100]. Abundant research suggests that miRNAs are involved in regulating endoplasmic reticulum (ER) stress adaptation. In osteosarcoma cells, ER stress-induced miR-1281 significantly induces cell apoptosis and reduces ER stress adaptation. Under ER stress, tumor suppressor p53 directly binds to the promoter of miR-1281 and promotes its expression. Evidence suggests that miR-1281, through targeting USP39, participates in ER stress-induced cell apoptosis. Thus, the p53-dependent miR-1281-mediated USP39 pathway inhibits the survival of human osteosarcoma cells under ER stress [100].

### 5.9. USP47

In osteosarcoma (OS) cells, high expression levels of DSCAM-AS1 promote cell proliferation, invasion, migration, and induce apoptosis. Molecular mechanism studies have confirmed that DSCAM-AS1 regulates the expression levels of USP47 by sponging miR-101-3p. It is noteworthy that knockdown of USP47 leads to inactivation of the Wnt-β-catenin signaling pathway and suppression of the AKT-mTOR signaling pathway [102]. Therefore, DSCAM-AS1 accelerates the progression of OS through the miR-101-3p-USP47 axis, suggesting its potential as a therapeutic target for OS treatment.

### 5.10. OTUB1

Containing the ovarian tumor domain (OTU), ubiquitin aldehyde binding protein 1 (OTUB1) is a member of the OTU superfamily of deubiquitinases, primarily involved in the progression of cancer and immune diseases [115]. In osteosarcoma U2OS cells, casein kinase 2 (CK2) phosphorylates OTUB1 at Ser16 of the deubiquitinase, leading to its phosphorylation without altering its catalytic activity but promoting its translocation from the cytoplasm to the nucleus. Phosphorylated OTUB1 in the nucleus facilitates the repair of DNA damage induced by ionizing radiation (IR) by promoting 53BP1 DNA repair foci formation [103].

### 5.11. UCHL1

Ubiquitin carboxyl-terminal hydrolase 1 (UCHL1) belongs to the UCH class of deubiquitinating enzymes (DUBs) and is primarily distributed in brain tissues, with lower expression in other normal tissues. There is limited research on UCHL1 in tumors, leaving ample room for exploration [116]. Zheng et al. [104] found that UCHL1 is significantly overexpressed in osteosarcoma tissues. Knockdown of the UCHL1 gene markedly inhibits the proliferation and invasion of osteosarcoma cells, and induces apoptosis in them. Results from in vivo tumor formation experiments in mice also demonstrate that knockout of the *UCHL1* gene suppresses the growth of osteosarcoma in nude mice. Importantly, the downregulation of UCHL1 leads to decreased levels of p-Akt and p-ERK, which may be a key mechanism by which UCHL1 exerts its oncogenic effect in osteosarcoma [104].

### 5.12. BAP1

BRCA1-associated protein-1 (BAP1) is a nuclear-localized deubiquitinase, expressed at low levels in osteosarcoma tissues and cells. BAP1 inhibits the proliferation, migration, and invasion of osteosarcoma cells by negatively regulating the PI3K/Akt signaling pathway. Additionally, miR-125 directly targets and binds to the 3′-UTR of the BAP1 transcript, thereby inhibiting BAP1 translation [105].

## 6. Ubiquilins

Ubiquilin 2 (UBQLN2), a member of the ubiquilin protein family, plays a role in maintaining dynamic protein balance. Tsukamoto et al. [117] demonstrated that UBQLN2 is highly expressed in osteosarcoma tissues and closely associated with a poor prognosis in patients. Under hypoxic conditions, knockdown of UBQLN2 leads to activation of JNK and p38, inducing apoptosis in the osteosarcoma cell line MG63. Moreover, injection of UBQLN2 siRNA inhibited tumor growth in a rat osteosarcoma model [117]. Ubiquitin-like with a plant homeodomain (PHD), RING-finger domain 1 (UHRF1) is aberrantly expressed in various human cancers, such as promoting the growth of liver cancer cells when overexpressed. Liu et al. found that UHRF1 enhances proliferation and invasion of human osteosarcoma cells in a retinoblastoma protein 1 (Rb1)-dependent manner. Specifically, Uhrf1 negatively regulates the mRNA and protein expression levels of Rb1, thereby inhibiting E-cadherin expression and ultimately promoting epithelial-to-mesenchymal transition (EMT) [118]. HLA-F locus adjacent transcript 10 (FAT10) is a member of the ubiquitin-like protein family, highly expressed in osteosarcoma cells [119,120,121], with its expression levels being more pronounced in metastatic osteosarcoma. Downregulation of FAT10 expression suppresses the invasive and migratory abilities of osteosarcoma cells, potentially through its positive regulation of β-catenin and HOXB9 expression to promote osteosarcoma development [120]. Additionally, research indicates that FAT10 interacts with YAP1 and promotes the ubiquitination and degradation of YAP1. The inhibitory effect on osteosarcoma cell proliferation caused by FAT10 knockout can be reversed by YAP1 overexpression. Therefore, FAT10′s regulation of osteosarcoma cell growth also depends on YAP1 [121]. Furthermore, elevated levels of FAT10 are closely associated with increased malignancy and reduced survival time in osteosarcoma patients. Biological behavior analysis suggests that FAT10 promotes osteosarcoma cell proliferation by targeting the glycolysis pathway. At the molecular level, FAT10 directly binds to EGFR and inhibits its ubiquitination and degradation, stabilizing the expression of EGFR and upregulating the protein level of PFKFB3, thereby enhancing glycolysis [119]. Small ubiquitin-like modifier (SUMO)-specific protease 2 (SENP2) is significantly underexpressed in OS tissue compared to adjacent normal tissue [122]. Overexpression of SENP2 inhibits the proliferation, migration, and invasion of OS cells, while knockdown of SENP2 has the opposite effect. Mechanistically, SENP2 promotes the proteasomal ubiquitination and degradation of SRY-box-9 (SOX9). Additionally, knockdown of SOX9 reverses the accelerated cell growth and migration caused by SENP2 depletion. Thus, the tumor suppressor gene SENP2 in osteosarcoma cells affects cell proliferation, migration, and invasion by targeting SOX9 [122]. MLN4924 is an inhibitor of NEDD8-activating enzyme (NAE), which in osteosarcoma reduces the ubiquitination and stabilizes the expression of the cullin substrate retinoid orphan nuclear receptor alpha (RORα), thereby antagonizing BMAL1 activation, inducing growth inhibition, and G2/M cell cycle arrest in U2OS cells [123]. Moreover, MLN4924 induces accumulation of several tumor-suppressive substrates of cullin-RING E3 ubiquitin ligases (CRLs), leading to DNA damage in OS cells, resulting in decreased cell viability, accelerated cellular senescence, and apoptosis [79] (see the previous section on E2 enzymes.).

## 7. Signaling Pathways

The table summarizes the four main signaling pathways involved in the ubiquitination process in osteosarcoma and the molecules associated with these pathways (see Table 2). The Wnt-β-catenin pathway and the molecules involved in it have been mentioned earlier and will not be reiterated here.

1. p53 pathway

The regulatory role of MDM2 on P53 has been extensively described in the previous text. In addition to this, P53 chimeras containing Gly-Ala repeat (GAr) domains of different lengths and positions within the protein are protected from proteolysis induced by the ubiquitin ligases MDM2 and E6-associated protein (E6-AP), but they are still ubiquitinated and retain the capacity to interact with the S5a ubiquitin-binding subunit of the proteasome. These P53 chimeras retain the ability to transcriptionally activate P53 target genes, inducing cell cycle arrest and apoptosis [124]. The human topoisomerase I- and p53-binding protein topors functions in mediating P53 ubiquitination, and overexpressed topors results in a proteasome-dependent decrease in p53 protein expression in OS cells [125]. Analysis of osteosarcoma (OS) patient datasets from the Gene Expression Omnibus (GEO) reveals significant involvement of cellular processes related to autophagy and protein processing in OS development. FDA-approved drugs chloroquine (CQ) and bortezomib (VP), known for their inhibition of autophagy and proteotoxicity, respectively, were investigated. Evidence suggests that VP, rather than CQ, demonstrates dose-dependent cytotoxicity extensively. Proteolytic products upon VP treatment exhibit a reactive oxygen species (ROS)-dependent high molecular weight (HMW) band when detecting p62 and p53 proteins. Furthermore, VP triggers the accumulation of ubiquitinated proteins. VP profoundly disrupts cellular protein homeostasis, with the addition of proteasome inhibitor MG132 significantly exacerbating VP-induced cytotoxicity. Combined therapy with MG132 also results in P53 selectively targeting lysosomes [126].

2. STAT pathway

FLLL32 is a novel compound synthesized from the naturally occurring phenolic compound curcumin, specifically targeting STAT3. STAT3 is a transcription factor that plays a significant role in tumor cell survival, proliferation, metastasis, and chemotherapy resistance. In OSA cell lines, the use of FLLL32 reduces STAT3 DNA binding and degrades STAT3 through the ubiquitin–proteasome pathway. Functionally, FLLL32 inhibits cell proliferation and promotes caspase-3-dependent apoptosis [127]. PARK2 is widely expressed in various tissues, encoding an E3 ubiquitin ligase involved in proteasome-mediated protein degradation [134]. PARK2 deficiency occurs in 30% of human malignancies [135]. A recent study demonstrates low expression of PARK2 in OS tissues and cells, correlating with advanced tumor stages. As a potential candidate tumor suppressor gene, the overexpression of PARK2 leads to cell cycle arrest, accelerated apoptosis, reduced cell proliferation, migration, invasion capabilities, and decreased angiogenesis. Mechanistic studies suggest that PARK2 inhibits OS progression through modulation of the JAK2/STAT3/VEGF pathway [128]. Geranylgeranylacetone (GGA) is an inducer of heat shock proteins with anticancer activity in various tumors. A study found that GGA inhibits OS cell proliferation in a dose-dependent manner and induces apoptosis. Specifically, GGA promotes the degradation of PRMT1 through the Hsp70-CHIP mediated proteasome pathway, inhibiting STAT3 methylation and activity, thereby inducing Fas-triggered apoptosis [129]. Collagen type VI alpha 1 (COL6A1) has been found to be dysregulated in several human malignancies. Zhang et al. [130] demonstrated upregulation of COL6A1 in OS tissues, closely associated with high pulmonary metastasis rates and poor prognosis of osteosarcoma. COL6A1 directly binds to SOCS5 and inhibits the expression and activation of STAT1 via ubiquitin–proteasome degradation, promoting the migration and invasion of OS cells [130].

3. PI3K/Akt Pathway

As mentioned earlier, molecules such as UBE2T, E3 ubiquitin ligase adaptor SPOP, USP9X, USP22, and BAP1 are involved in regulating the proliferation, migration, and invasion of osteosarcoma cells through modulation of the PI3K/Akt signaling pathway [53,93,97,105]. In addition, Zong et al. found that CSN5 is highly expressed in osteosarcoma (OS), and its high expression level is significantly correlated with the malignant phenotype and poor prognosis of OS patients. Mechanistically, CSN5 inhibits the ubiquitination level of EGFR and stabilizes EGFR protein expression, further activating the PI3K/Akt signaling pathway to promote the growth of OS cells [131]. Li et al. [132] analyzed differentially expressed genes between circulating tumor cells (CTCs) and osteosarcoma metastases using bioinformatics tools, and the results indicated that TRAIP might be a prognostic-related gene for osteosarcoma. Experimental results showed that TRAIP downregulates the expression of IGFBP3 by promoting the ubiquitination and degradation of KANK1, thereby activating the AKT pathway and promoting the proliferation and invasion of osteosarcoma cells [132]. IRS4 is a member of the insulin receptor substrate protein family, inducing constitutive PI3K/AKT hyperactivation and cell proliferation, thereby promoting tumorigenesis. In osteosarcoma cells and tissue samples, the protein levels of CK1γ2 and IRS4 are negatively correlated. IRS4 is phosphorylated by CK1γ2 at Ser859 both in vitro and in vivo, promoting its ubiquitination and degradation through the carboxyl terminus of Hsc70-interacting protein (CHIP) interaction. It is noteworthy that in osteosarcoma cell lines, the CK1γ2-induced non-phosphorylatable mutant of IRS4 exhibits higher levels of p-Akt both in vitro and in nude mice, showing faster cell proliferation and tumor growth [133].

## 8. Other Related Studies

PPI, derived from Paris root, represents a natural compound found in traditional Chinese herbal medicine, known for its antipyretic and detoxifying properties. Zhang et al. [136] found that PPI selectively inhibits the CT-like (chymotrypsin-like) proteasomal activity in purified human proteasomes and cultured osteosarcoma cell proteasomes, leading to the accumulation of ubiquitinated proteins. This accumulation induces apoptosis and cell cycle arrest in osteosarcoma cells, and reverses epithelial–mesenchymal transition (EMT), demonstrating effective anti-osteosarcoma activity [136]. Rev1 is a DNA damage tolerance protein that encodes two ubiquitin-binding motifs (UBM1 and UBM2), playing a crucial role in cell survival under DNA damage stress. In osteosarcoma U2OS cells, the small molecule compound MLAF50 directly interacts with Rev1 UBM2, weakening the interaction between Rev1 UBM2 and ubiquitin and preventing the chromatin localization of Rev1 induced by cisplatin in U2OS cells [137], suggesting that Rev1 UBM2 may serve as a target for small molecule inhibitors for the treatment of osteosarcoma. MAS-related G protein-coupled receptor C subtype (MRGC) ubiquitination plays a crucial role in bone cancer pain. Sun et al. found that MRGC ubiquitination is involved in the generation and maintenance of bone cancer pain in mice, possibly through regulating calcium ion concentration within mouse spinal neurons [138]. Additionally, the table below presents other relevant studies of the ubiquitination system in osteosarcoma (Table 3).

## 9. Conclusions

In the treatment of osteosarcoma, surgical resection combined with chemotherapy is currently the main treatment modality. In the selection of chemotherapy drugs, MAP (high-dose methotrexate, doxorubicin/cisplatin, and cisplatin) is the most widely used and effective chemotherapy regimen for OS [153,154]. However, the treatment outcomes for recurrent or metastatic OS patients remain unsatisfactory. Results from several clinical trials indicate that adjuvant chemotherapy (with or without high-dose ifosfamide in combination with MAP) has limited efficacy in OS patients [155]. Additionally, the toxic side effects of chemotherapy and the presence of drug resistance significantly limit its application in osteosarcoma. Subsequently, analysis of the tumor microenvironment (TME) in OS revealed the infiltration of immune cells including macrophages and T cells [156], providing a basis for immunotherapy in osteosarcoma. However, the broad cytotoxic effects of immunotherapy on a wide range of cells continue to restrict its application. Currently, with the further development of medical technology, precision medicine is gradually becoming mainstream. Its core concept is to select the most suitable targeted drugs based on the patient’s molecular characteristics and genomic variations to achieve more precise, effective, and personalized treatment outcomes. The advantages of targeted drugs largely improve the therapeutic effects while reducing the toxic side effects on patients, bringing new hope for treatment. Currently, research targets related to osteosarcoma treatment mainly include DNA damage repair and cell cycle, vascular endothelial growth factor (VEGF), platelet-derived growth factor (PDGF), insulin-like growth factor (IGF), PI3K/mTOR pathway, avian myelocytomatosis viral oncogene homolog (MYC), among others, and numerous drugs corresponding to these targets have entered clinical trial stages [157].

The ubiquitin–proteasome system is an important mechanism that regulates protein levels and activity within cells. Almost every step of the ubiquitination process is involved in the occurrence and development of osteosarcoma as well as its impact on drug resistance mechanisms in osteosarcoma. The role of E3 ubiquitin ligases is particularly prominent in this process. Many molecules in the TRIM protein family participate in the occurrence and development of osteosarcoma through the role of E3 ubiquitin ligases; MDM2 and CRLs regulate the expression of P53 and P21 molecules to participate in the proliferation, migration, and invasion of osteosarcoma; RAD18 and RNF8 play a role in tumor drug resistance mechanisms by participating in DNA damage repair. In addition, deubiquitinating enzymes also play an important role in this process. The ubiquitination process not only involves multiple biological processes but also affects multiple cell signaling pathways. Therefore, designing and developing new drugs targeting the ubiquitin–proteasome system in osteosarcoma and addressing traditional drug resistance have significant clinical value and prospects. Furthermore, further research on the role of the ubiquitin–proteasome system in osteosarcoma mechanisms is expected to provide new insights and directions for osteosarcoma treatment and prognosis.

## Figures and Tables

**Table 1:** The involvement of various DUBs in the process of osteosarcoma and the signaling axes involved.

DUBs	Participating Process	Regulatory Axis
USP1	Maintaining osteosarcoma stem cell state [82]; Promoting osteosarcoma growth and invasion [83,84]; Involved in DNA damage response [85].	USP1/IDs [82]; USP1/TAZ/Hippo pathway [84]; miR-192-5p/USP1 [86]; USP1-UAF1 [85].
USP4/USP17	Promoting osteosarcoma cell proliferation, migration, and invasion [87,88].	(USP4/USP17)/PDGFRβ/STAT3 pathway [87]; USP17/SMAD4 [88].
USP7	Promoting osteosarcoma cell proliferation, migration, and invasion [89,90,91].	USP7/Wnt-β-catenin pathway [91]; USP7/AKT/GSK-3β/EMT [89]; E2/mTORC1/USP7/NLRP3 pathway [90].
USP9X	Promoting osteosarcoma cell proliferation, migration, and invasion [92,93].	USP9X-SOX2 [92]; USP9X/ERK1/2 [93]; USP9X/PI3K/Akt pathway [93].
USP11	Involved in DNA damage response, promoting HR repair [94]; Involved in bipolar spindle formation [95].	USP11/RAE1 [95].
USP22	Promoting osteosarcoma cell proliferation, migration, and invasion [96,97].	USP22/PI3K/Akt pathway [97]; USP22/EMT [97]; USP22/LSD1 [96]; MiR-140/USP22 [96] ALKBH5/USP22(m6A) [98]; linc00265/miR-485-5p/USP22 [99].
USP39	Promoting apoptosis in osteosarcoma cells and participating in cell cycle regulation [100,101].	USP39/p21 [100]; USP39/PARP [100]; miR-1281/USP39/ER stress [101].
USP47	Promoting osteosarcoma cell proliferation, migration, and invasion [102].	USP47/Wnt-β-catenin pathway [102]; USP47/AKT-mTOR pathway [102]; DSCAM-AS1/miR-101-3p/USP47 [102].
OTUB1	Involved in DNA damage repair [103].	CK2/OTUB1/53BP1 [103].
UCHL1	Promoting osteosarcoma cell proliferation and invasion, while inhibiting apoptosis [104].	UCHL1/p-Akt [104]; UCHL1/p-ERK [104].
BAP1	Inhibiting proliferation, migration, and invasion of osteosarcoma cells [105].	BAP1/PI3K-Akt pathway [105]; miR-125/BAP1 [105].

**Table 2:** The four main signaling pathways involved in the ubiquitination process in osteosarcoma and the molecules associated with these pathways.

Pathways	Molecules and Drugs	Regulation of Pathways by Molecules or Drugs	References
P53 pathway	MDM2	Negative regulation.	[42]
	P53 chimeras	Positive regulation.	[124]
	Topors	Negative regulation.	[125]
	VP+MG132	Negative regulation.	[126]
STAT pathway	USP4/USP17	Positive regulation (STAT3).	[87]
	FLLL32	Negative regulation (STAT3).	[127]
	PARK2	Negative regulation (STAT3).	[128]
	GGA	Negative regulation (STAT3).	[129]
	COL6A1	Negative regulation (STAT1).	[130]
PI3K/Akt pathway	UBE2T	Negative regulation.	[78]
	SPOP	Negative regulation.	[53]
	USP9X	Downregulation of USP9X inhibits the activation of the PI3K/Akt signaling pathway.	[93]
	USP22	Downregulation of USP22 inhibits the activation of the PI3K/Akt signaling pathway.	[97]
	BAP1	Negative regulation.	[105]
	CSN5	Positive regulation.	[131]
	TRAIP	Positive regulation.	[132]
	IRS4	Positive regulation.	[133]
Wnt-β-catenin pathway	SIAH1	Positive regulation.	[63]
	USP7	Positive regulation.	[91]
	USP47	Downregulation of USP47 results in the inactivation of the Wnt/β-catenin signaling pathway.	[102]

**Table 3:** Other relevant studies of the ubiquitination system in osteosarcoma.

Molecules	Processes	Mechanism	References
pRB	Regulation of the cell cycle.	Preventing the degradation of transcription factor E2F1 via the ubiquitin–proteasome pathway.	[139]
E2F1	Regulation of cell differentiation.	Specifically binding to RARα, promoting its ubiquitin-mediated degradation.	[140]
GRP78	Regulation of endoplasmic reticulum stress.	Directly interacting with the N-terminal domain of CHOP and promoting CHOP ubiquitination in a p300-dependent manner.	[141]
Gankyrin	Promoting migration and invasion.	“Interacting with Gli1 to inhibit ubiquitin-proteasome-mediated degradation of Gli1 and regulating the expression of stemness factors.”	[142]
EPIC1	Inhibiting invasion.	A long non-coding RNA that negatively regulates the expression of MEF2D protein by increasing its ubiquitination levels.	[143]
DBC1 and AR	Involved in proliferation and proliferation-related signaling pathways.	DBC1 negatively regulates AR protein expression by inhibiting AR polyubiquitination–proteasome pathway degradation.	[144]
PVT1	Promoting proliferation and migration.	By inhibiting the ubiquitination of ERG and competitively binding with miR-183-5p, leading to upregulation of ERG expression.	[145]
ROCK2	Promoting proliferation, migration, and invasion; Participating in TRAIL resistance and inhibiting apoptosis.	ROCK2 interacts directly with PFKFB3, reducing the ubiquitination level of PFKFB3 and increasing its stability; ROCK2 inhibits TRAIL activation and osteosarcoma cell apoptosis by ubiquitin–proteasome degradation of OGT.	[146,147]
HDAC4 and PCNA	Promoting proliferation and invasion while inhibiting apoptosis.	HDAC4 directly binds to PCNA and inhibits PCNA ubiquitination–proteasome degradation, positively regulating PCNA protein expression.	[148]
FAM83H	Promoting proliferation and invasive capacity.	FAM83H inhibits the ubiquitination–proteasome degradation of β-catenin by interacting with it.	[149]
miR-221	Promoting proliferation and inhibiting cell apoptosis.	Inhibiting the Wnt signaling pathway by targeting the ubiquitin–proteasome system component FBXW11.	[150]
CircECE1	Promoting proliferation and metastasis.	Interacting with c-Myc, preventing SPOP-mediated c-Myc ubiquitination and degradation, thereby activating the Warburg effect through TXNIP transcription.	[151]
LAMTOR5-AS1	Inhibiting proliferation and multidrug resistance	Mediating the interaction between NRF2 and KEAP1 inhibits the ubiquitination degradation of NRF2, upregulating the expression of HO-1.	[152]
